# An *APETALA2* Homolog, *RcAP2*, Regulates the Number of Rose Petals Derived From Stamens and Response to Temperature Fluctuations

**DOI:** 10.3389/fpls.2018.00481

**Published:** 2018-04-12

**Authors:** Yu Han, Aoying Tang, Huihua Wan, Tengxun Zhang, Tangren Cheng, Jia Wang, Weiru Yang, Huitang Pan, Qixiang Zhang

**Affiliations:** ^1^Beijing Key Laboratory of Ornamental Plants Germplasm Innovation & Molecular Breeding, National Engineering Research Center for Floriculture, Beijing Laboratory of Urban and Rural Ecological Environment, Key Laboratory of Genetics and Breeding in Forest Trees and Ornamental Plants of Ministry of Education, School of Landscape Architecture, Beijing Forestry University, Beijing, China; ^2^Beijing Advanced Innovation Center for Tree Breeding by Molecular Design, Beijing Forestry University, Beijing, China

**Keywords:** *APETALA2*, *Rosa chinensis*, petals derived from stamens, petal number, temperature fluctuations

## Abstract

*Rosa chinensis*, which is a famous traditional flower in China, is a major ornamental plant worldwide. Long-term cultivation and breeding have resulted in considerable changes in the number of rose petals, while most wild Rosaceae plants have only one whorl consisting of five petals. The petals of double flowers reportedly originate from stamens, but the underlying molecular mechanism has not been fully characterized. In this study, we observed that the number of petals of *R. chinensis* ‘Old Blush’ flowers increased and decreased in response to low- and high-temperature treatments, respectively, similar to previous reports. We characterized these variations in further detail and found that the number of stamens exhibited the opposite trend. We cloned an *APETALA2* homolog, *RcAP2*. A detailed analysis of gene structure and promoter *cis*-acting elements as well as *RcAP2* temporospatial expression patterns and responses to temperature changes suggested that *RcAP2* expression may be related to the number of petals from stamen origin. The overexpression of *RcAP2* in *Arabidopsis thaliana* transgenic plants may induce the transformation of stamens to petals, thereby increasing the number of petals. Moreover, silencing *RcAP2* in ‘Old Blush’ plants decreased the number of petals. Our results may be useful for clarifying the temperature-responsive mechanism involved in petaloid stamen production, which may be relevant for the breeding of new rose varieties with enhanced flower traits.

## Introduction

The China rose (*Rosa chinensis*), which is one of the most popular ornamental plants, produces flowers with diverse patterns. The considerable ornamental value of *R. chinensis* is at least partly due to the variable double-flower trait with different colors. Double-flower trait describes varieties of flowers with extra petals, and is the earliest documented form of floral abnormality, which was first recognized 2,000 years ago (Meyerowitz et al., 1989). Most wild Rosaceae species have only one whorl of five petals, while *R. chinensis* produces a double flower, which may originate from the stamen (Reynolds and Tampion, 1983). These petals evolved from the stamens, with the filaments widening to form narrow petals (Kramer and Irish, 1999; Cronk, 2001). A study of a rose hybrid population revealed that the double-flower phenotype is a dominant qualitative trait that is controlled by a single gene (Debener, 1999). However, the degree of double flowering, that is, the number of petals produced by secondary-effect genes, has not been fully characterized (Morey, 1959; Hibrand-Saint Oyant et al., 2008). In addition to genetic factors, environmental cues (e.g., temperature) also affect the number of petals (Garrod and Harris, 1974; Chmelnitsky et al., 2001). Low temperatures increase the number of petals and decrease the number of stamens, resulting in the bullhead rose phenotype (Zieslin, 1968, 1992). The development of petals from stamen origin represents the main factor involved in increasing the number of rose petals. The underlying mechanism is likely relatively complex.

Elucidating the mechanism regulating petal formation is one of the most important objectives of floral organ research. The floral organ model has been extensively studied, and the ABCE model has gradually been accepted (Soltis et al., 2006). This model adopts the basic theory of the classical ABC model, while simultaneously emphasizing the importance of the class E genes regarding floral organ development. Specifically, the class E genes are expressed in the four whorls of floral organs, and are indispensable in the development of sepals, petals, stamens, and carpels, although there is some functional redundancy. In the ABCE model, class A + E genes determine the formation of sepals, class A + B + E genes mediate the formation of petals, class B + C + E genes contribute to the formation of stamens, and class C + E genes are involved in the formation of carpels (Bowman et al., 1991; Coen and Meyerowitz, 1991; Causier et al., 2010). Several homeotic genes have been identified in each class, including *APETALA1* (*AP1*) and *APETALA2* (*AP2*) in class A, *APETALA3* (*AP3*) and *PISTILLATA* (*PI*) in class B, *AGAMOUS* (*AG*) in class C, and *SEPALLATA1* (*SEP1*), *SEP2*, *SEP3*, and *SEP4* in class E (Pelaz et al., 2000). Based on an analysis of flower morphology in dicotyledonous plants, such as buttercup, and monocotyledons, including lily and tulip, the shifting border model has been proposed (Van Tunen et al., 1993; Bowman, 1997; Kramer et al., 1998). In this model, the tissues in which the class A and C genes are expressed are relatively fixed, but because of the expansion of the class B genes, the outer sepals can develop into petals (Kanno et al., 2007). The expansion of the class B genes has also been observed in other dicotyledonous plants, such as *Sagittaria* and *Camellia* species (Kramer et al., 1998). Moreover, recent studies regarding *Macleaya* and Rosaceae species have revealed that the tissues in which the class A and C genes are expressed may be variable (Ronse De Craene, 2003; Dubois et al., 2010). However, there are relatively few examples of a change in the tissues in which class A genes are expressed. Although the shifting border model can complement the ABCE model to some extent, it should be studied in greater detail. For example, additional floral organ development genes and plant species should be investigated to further characterize the role of the shifting border model during floral organ development.

Most of the studies on the double-flower phenotype have focused on the class C genes. In *Arabidopsis thaliana*, AG regulates stamen and carpel properties. The stamens are transformed into petals to form double flowers in the *ag* mutant. Earlier investigations confirmed that AG interacts with the transcription factor WUSCHEL (WUS), which controls the differentiation of the meristem, and then determines the floral organ properties (Lenhard et al., 2001; Lohmann et al., 2001). A previous study indicated that the Japanese gentian double-flower mutant is the result of the insertion of a 5,150-bp LTR retrotransposon in the sixth intron of *GsAG1* (Nakatsuka et al., 2015). Meanwhile, the double-flower phenotype of *Prunus lannesiana* is due to the absence of a *PrseAG* exon (Liu et al., 2013). Additionally, the *RhAG* (*Rosa hybrida* cv. Vendela) expression level is low in the double flowers of cultivated rose species. The results of an *in situ* hybridization indicated that *RhAG* expression is concentrated in the central region of meristematic tissue, resulting in an increase in the number of petals and a decrease in the number of stamens (Dubois et al., 2010). Additional research confirmed that exposure to low temperatures alters the methylation level of a specific region of the *RhAG* promoter to regulate expression (Ma et al., 2015).

The mechanism that determines the number of petals has not been fully characterized, especially in rose species. First, *RhAG* is not located at the *Blfo* locus, the marker closest to the gene for double flowers (Debener and Mattiesch, 1999; Dugo et al., 2005), which may control whether single or double flowers are produced, or at the locus associated with the number of petals (Spiller et al., 2011). This suggests that *RhAG* may function as a downstream regulatory gene (Dubois et al., 2010). Second, the restricted spatial expression of *RhAG* may be insufficient for the transition of stamens to petals, necessitating the participation of class A genes (Spiller et al., 2011). The third whorl of the floral organ develops as stamens or petals depending on the balance between *AP2* and *AG* (Wollmann et al., 2010). In *A. thaliana*, *AP2* determines the sepal and petal characteristics, while limiting the tissues in which the class C genes are expressed, and regulating *AG* expression to upregulate the expression of class B genes (Liu and Meyerowitz, 1995; Jack, 2004). A lack of *AP2* function will lead to stamens replacing petals, while the ectopic expression of *AP2* will result in the production of double flowers (Theissen et al., 2000). Thus, the regulatory effect of *AP2* on the number of rose petals cannot be ignored.

‘Old Blush’ is a diploid *R. chinensis* cultivar that originated in China over 1,000 years ago. This rose cultivar exhibits recurrent flowering and produces double flowers as well as other traits that are very important for modern rose breeding (Dubois et al., 2012). We observed that the number of petals in ‘Old Blush’ flowers differs considerably depending on whether plants are exposed to low or high temperatures. We identified an *AP2* homolog (*RcAP2*) in ‘Old Blush’ based on previous studies. This homolog has an alternative splice variant (*RcAP2v*). We completed detailed analyses of genetic structures and subcellular localization. We also determined that the *RcAP2* expression pattern is correlated with petal type and the degree of double flowering, especially under low or high temperatures. Additionally, *RcAP2* was heterologously expressed and transiently silenced in *A. thaliana* and rose, respectively. Our results may help to clarify the regulatory mechanism associated with rose floral organ formation and contribute to the molecular breeding of rose species for the production of desirable flower types.

## Materials and Methods

### Plant Materials and Growth Conditions

*Rosa chinensis* ‘Old Blush’ and *A. thaliana* ‘Columbia’ plants were grown in a greenhouse under a 12-h light [photosynthetic photon flux density (PPFD) of 800 μmol m^-2^ s^-1^]: 12-h dark photoperiod. The plants were incubated at 24°C (light): 14°C (dark) with 60% relative humidity. Rose flower development was divided into 11 stages based on the phenotype as well as the results of an earlier analysis of paraffin sections (Jackson, 1991). The major developments at each stage were as follows: S1: vegetation cone and leaf primordia formed; S2: floral meristem formed; S3: sepal primordia appeared; S4: petal primordia appeared and sepal primordia continued to develop; S5: stamen primordia appeared and developed; S6: pistil primordia appeared and other types of primordia continued to develop; S7: small buds appeared (approximately 2 mm diameter); S8: small buds increased in size (approximately 4 mm diameter); S9: buds differentiated, but sepals remained intact; S10: flowers started to open; and S11: flowers were fully open, with carpels and stamens visible. All materials were collected at 12:00 am (25°C). Ten buds were collected at S1–S6, while six buds or flowers were harvested at S7–S11. The collected samples were pooled and immediately frozen in liquid nitrogen. Sample collections were completed with three biological replicates.

### RNA and DNA Extraction

Total RNA was extracted using the SV Total RNA Isolation System (Promega, Madison, WI, United States), and used as the template for the first-strand cDNA synthesis using the PrimeScript^TM^ RT Reagent Kit with gDNA Eraser (Takara Bio Inc., Shiga, Japan). Additionally, DNA was extracted from *R. chinensis* ‘Old Blush’ leaves using the Plant Genomic DNA kit (Omega Bio-tek Inc., Norcross, GA, United States).

### Gene and Promoter Isolation

Primers *RcAP2*-F1 and *RcAP2*-R1 were designed based on the conserved sequences of *AP2* homologs to PCR amplify the *RcAP2* gene. The cDNA samples appropriate for rapid amplification of cDNA ends (RACE) were synthesized using the SMARTer^TM^ RACE cDNA Amplification Kit (Clontech, United States). The 5′-GSP primers for the 5′-RACE were designed according to the sequences obtained from the 3′-RACE experiment. The Universal Primer Mix from the 5′- and 3′-RACE PCR kit was used for a touchdown PCR, which was completed with the following program: five cycles of 94°C for 30 s and 72°C for 3 min; five cycles of 94°C for 30 s, 70°C for 30 s, and 72°C for 3 min; 27 cycles of 94°C for 30 s, 68°C for 30 s, and 72°C for 3 min. Primers *RcAP2*-F2 and *RcAP2*-R2 were designed based on the 5′- and 3′-ends of the candidate sequence, and used to amplify the full-length gene with the following program: 94°C for 5 min; 35 cycles of 94°C for 30 s, 56°C for 30 s, and 72°C for 3 min; 72°C for 10 min. The *RhAG* sequence (RHU43372) was used to design primers *RcAG*-F and *RcAG*-R for the amplification of the *RcAG* DNA sequence. The *RcAP2* promoter was obtained using the Genome Walking Kit (Takara). Details regarding primers *proRcAP2*-SP1, *proRcAP2*-SP2, and *proRcAP2*-SP3 are provided in Supplementary Table S1. Amplified cDNA or promoter fragments were purified with the MiniBEST Agarose Gel DNA Extraction Kit (Takara). The purified products were ligated into the pEASY-blunt cloning vector (TransGen Biotech, China) and subsequently transformed into *Escherichia coli* DH5α cells. Positive colonies were sequenced by SinoGenoMax (China). Details regarding the *RcAP2* CDS (GenBank ID KX371592), DNA sequence (GenBank ID MF773427), splice variants (GenBank ID MF773425), and promoter sequence as well as the *RcAG* sequence (GenBank ID MF773426) have been deposited in the NCBI database^1^. Details regarding all primers are provided in Supplementary Table S4.

### Bioinformatics Analysis

Sequence analysis and contig assembly were completed using DNAMAN software, while the *RcAP2* open reading frame (ORF) was predicted using ORF Finder^2^. Multiple sequences were aligned using ClustalX with default parameters. The alignments were edited and marked using GeneDoc software. Phylogenetic analyses were conducted with MEGA7 using the Neighbor-Joining method and the bootstrap consensus tree was inferred from 1000 replicates. The function prediction of transcription factor using PlantTFDB online tool^3^, the RegRNA 2.0 online tool^4^ (Chang et al., 2013) was used to identifying the functional motifs and sites of *RcAP2*. The *cis*-acting element analyses were completed using online software PlantCARE^5^ (Lescot et al., 2002) and SoftBerry^6^ (Solovyev et al., 2010).

The GenBank ID of AP2 protein sequences showed in alignment analyses were as follows: AAL57045.2 *Malus domestica*; AKN10311.1 *Eriobotrya japonica*; AJT39804.1 *Prunus mume*; AEB92231.1 *Prunus persica*; ACO52508.1 *Vitis vinifera*; EOX91494.1 *Theobroma cacao*; AEL29576.1 *Betula platyphylla*; EXC24730.1 *Morus notabilis*; AGM20693.1 *Populus tomentosa*; EEE91165.1 *Populus trichocarpa*; KYP44918.1 *Cajanus cajan*; KHN36247.1 *Glycine soja*; AIS71911.1 *Nicotiana tabacum*; and AAC13770.1 *Arabidopsis thaliana*.

The GenBank ID of *AP2* sequences showed in phylogenetic analyses were as follows: HQ637468.1 *Brassica napus*; JN247401.1 *Actinidia deliciosa*; JN247408.1 *Betula platyphylla*; JF683605.2 *Prunus persica*; U12546.1 *Arabidopsis thaliana*; DQ988341.1 *Larix marschlinsii*; AB495344.1 *Nymphaea hybrid*; JQ398741.1 *Camellia sinensis*; HQ113365.1 *Actinidia deliciosa*; EU883665.1 *Poncirus trifoliata*; FJ809943.1 *Vitis vinifera*; DQ988340.1 *Larix marschlinsii*; KP405836.1 *Prunus mume*; AB495343.1 *Nymphaea hybrid*; AF134116.3 *Hyacinthus orientalis*; AY128328.1 *Arabidopsis thaliana*; AY069953.1 *Hordeum vulgare*; AF253971.1 *Picea abies*; AF253970.1 *Picea abies*; EU677382.1 *Solanum lycopersicum*; KT163121.1 *Thermopsis* textitturcica; KJ482645.1 *Brassica juncea*; EF216863.2 *Ipomoea nil*; DQ119837.1 *Dendrobium crumenatum*; AB195246.1 *Gnetum parvifolium*; AB195244.1 *Ginkgo biloba*; AB195242.1 *Cycas revoluta*; AB101587.1 *Pinus thunbergii*; AB101586.1 *Pinus thunbergii*; KM386630.1 *Fagopyrum esculentum*; KM386629.1 *Fagopyrum esculentum*; KM386628.1 *Fagopyrum esculentum*; and GU983666.1 *Malus domestica*.

### qPCR Validation

The qPCR assay was completed with the following program: 95°C for 30 s; 40 cycles of 95°C for 5 s and 60°C for 30 s; 95°C for 15 s, 60°C for 1 min and 95°C for 15 s for the melting-curve analysis. The qPCR solution consisted of 2 μL first-strand cDNA, 10 μL SYBR Premix Ex Taq (Takara Bio Inc., Shiga, Japan), 0.4 μL 10 μM forward and reverse primers, and 7.2 μL sterile distilled water. Each sample was assessed with three technical replicates. The expression data were normalized using *RcActin* or *AtActin* as the internal controls according to the 2^-ΔΔCt^ method (Young et al., 2010). The qPCR primers are listed in Supplementary Table S4. The qPCR assay was conducted with three biological replicates. Gene expression diagrams were prepared with the Origin 8 program (OriginLab, Northampton, MA, United States).

### Temperature Treatments of *Rosa chinensis* ‘Old Blush’

Two-year-old ‘Old Blush’ plants grown in pots under the conditions described in the Section “Plant materials and growth conditions” were used to determine the effects of temperature on the number of rose petals. Two artificial climate chambers were set as follows: 800 μmol m^-2^ s^-1^ PPFD; 12-h light: 12-h dark cycle; 60% relative humidity; and one of two temperature regimens [i.e., 16°C (light): 6°C (dark); 24°C: 14°C; or 32°C: 22°C]. Each temperature treatment involved 30 rose plants. The samples which collected at 24°C: 14°C for *RcAP2* expression analysis were as control, and using the same data for indicating *RcAP2* expression levels at S1–S11 and comparing with high-or low-temperature treatments. All buds at the beginning of S1 were observed. The buds and flowers collected at all floral development stages were frozen in liquid nitrogen until analyzed. We counted the number of floral organs (i.e., sepals, petals, stamens, and carpels) at S11. Twenty randomly selected flowers were examined for each treatment. The petals derived from stamens were considered as petals. Significant differences were determined according to Fisher’s LSD (*P* < 0.05) and Student’s *t*-test.

### Heterologous Expression in *Arabidopsis thaliana*

The *RcAP2* cDNA sequence was amplified using primers *RcAP2*-F3 and *RcAP2*-R3 (Supplementary Table S4), and the PCR product was cloned into the *Sal*I/*Spe*I-cleaved pSuper1300 vector using the In-Fusion^®^ HD Cloning Kit (Clontech, United States). The resulting pSuper1300-RcAP2 vector, in which *RcAP2* was under the control of the cauliflower mosaic virus *35S* promoter, was introduced into *A. thaliana* (Columbia) cells according to the floral-dip method involving *Agrobacterium tumefaciens* EHA105 (Clough and Bent, 1998). The T_0_ seeds were selected on MS medium containing 50 mg/L hygromycin B (Roche, Germany). The positive lines were confirmed by RT-PCR using primers RT-*RcAP2*-F/R and RT-*AtActin*-F/R. Choose three lines with highest expression of *RcAP2* and identified their T_3_ pure lines with *RcAP2* high expression levels using RT-PCR and qPCR methods. The floral phenotypes of T_3_ transgenic plants were observed and the flowers were collected. Ten flowers were analyzed, with three technical replicates. Significant differences were determined according to Fisher’s LSD (*P* < 0.05) and Student’s *t*-test.

### *RcAP2* Silencing in ‘Old Blush’ Plants

To silence *RcAP2* in ‘Old Blush’ plants, a 405-bp *RcAP2* CDS fragment was amplified using primers pTRV2-*RcAP2*-F/R (Supplementary Table S4). The PCR product was cloned into the *BamH*I/*EcoR*I-cleaved TRV2 vector (Liu et al., 2002) using the In-Fusion^®^ HD Cloning Kit (Clontech, United States). The pTRV1, pTRV2, and pTRV2::*RcAP2* plasmids were inserted into *A. tumefaciens* AGL0 cells. The silencing of *RcAP2* was achieved using a graft-accelerated VIGS method (Yan et al., 2017). Thirty flowers bloomed after the *A. tumefaciens*-mediated insertion of pTRV1 + pTRV2 or pTRV1 + pTRV2::*RcAP2*. The floral phenotypes of the transgenic plants were observed and the flowers were collected. In pTRV1 + pTRV2::*RcAP2* treatments, twelve sprouts appeared petal reduction phenotype after flowering. Each four flowers mixed-samples as a line for phenotypes observed and expression analysis. Samples were frozen in liquid nitrogen for a subsequent qPCR analysis of *RcAP2* expression.

### Subcellular Localization of RcAP2

The RcAP2 cDNA sequence was amplified using primers *RcAP2*-F4 and *RcAP2*-R4. The PCR product was cloned into the *EcoR*I/*Hind*III-cleaved pEZS-NL transient expression vector (provided by D. Ehrhardt, Carnegie Institution, Stanford, CA, United States). To examine the subcellular localization of RcAP2, isolated maize protoplasts were transformed with the prepared vector using an established protocol (Sheen et al., 1995). The transformed protoplasts were incubated for 16 h and then stained with 0.1 mg/mL 4′,6-diamino-2-phenylindole (DAPI; Sigma-Aldrich) in phosphate-buffered saline for 10 min. The RcAP2 protein was localized based on GFP fluorescence, which was detected with a laser confocal microscope (Leica TCS SP8, Germany). The GFP and DAPI were excited at 488 and 408 nm, respectively.

## Results

### *RcAP2* Is an Ortholog of *APELATA2* and Has an Alternative Splice Variant (*RcAP2v*)

**FIGURE 1:**
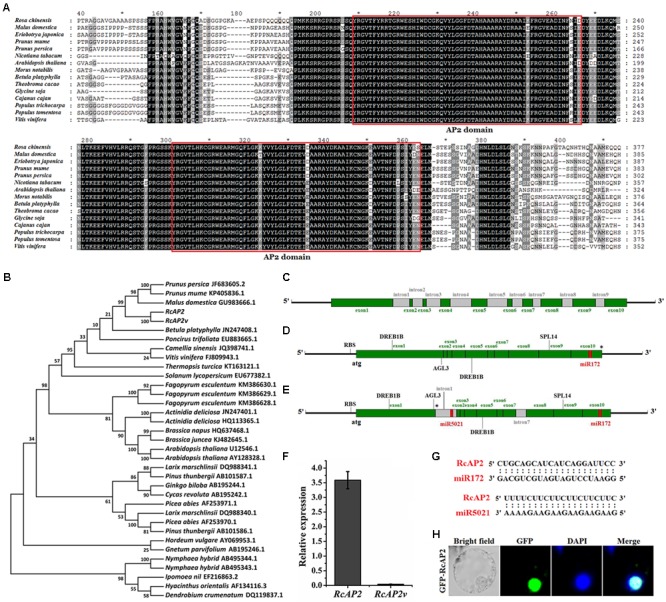
Bioinformatic analysis of RcAP2. **(A)** Sequence alignments of AP2 amino acid sequences among 15 different species. Deduced amino acid sequences of RcAP2 and another 14 AP2s were aligned using ClustalX with default settings and edited using GeneDoc. Boxes show two predicted AP2 domains. Each asterisk in **(A)** indicated the middle position of its left and right numbers. **(B)** Phylogenetic analysis of AP2 homologs from various species. The phylogenetic trees were constructed using MEGA7 software. The GenBank ID of each AP2 has been labeled. **(C)** Gene structure of *RcAP2* DNA sequence. Green boxes, exons; gray boxes, introns. **(D)** Gene structure diagram of *RcAP2* cDNA sequence. Four functional motifs (two DREB1B, one AGL3, and one SPL14) and one miR172 binding site were identified using RegRNA 2.0 online tool. RBS, ribosomal binding site. Asterisk marks termination codon. **(E)** Gene structure of *RcAP2v* cDNA sequence. Gray boxes, introns. One miR5021 binding site is shown in red. RBS, ribosomal binding site. Asterisk marks termination codon. **(F)** qPCR analysis of *RcAP2* and *RcAP2v* transcript levels in whole flowers at 24°C (light): 14°C (dark). **(G)** Sequence alignments of two predicted miRNA-binding sequences and *RcAP2*. **(H)** Subcellular localization of RcAP2. RcAP2 and GFP fusion protein localized to nucleus of maize protoplasts. Bars = 10 μm.

To identify the *R. chinensis AP2* ortholog, we obtained the *RcAP2* DNA and mRNA sequences as well as the sequences of the promoter and a splice variant, *RcAP2v*. The encoded RcAP2 protein, which consists of 535 amino acids, was aligned with the AP2 sequences from 13 different species, all of which contained two conserved AP2 domains (**Figure 1A**). We constructed a phylogenetic tree based on the full-length cDNA sequences to assess the relationships among *RcAP2* and the other *AP2* genes available in the NCBI database (**Figure 1B**). The *R. chinensis AP2* was most closely related to the *AP2* genes from *Prunus persica*, *Prunus mume*, and *Malus domestica* (i.e., Rosaceae species), followed by the *Betula platyphylla AP2* gene.

*RcAP2* contains 10 exons and 9 introns (**Figure 1C** and Supplementary Table S1). When we cloned the *RcAP2* full-length coding sequence (CDS) from rose cDNA, we also obtained a CDS that comprised the first and seventh intron. This additional CDS may represent an intron retention splice variant of *RcAP2* (**Figure 1E**). We then predicted the transcriptional regulatory motifs and miRNA-binding sites using the RegRNA 2.0 online tool. Four functional motifs were detected in *RcAP2* and *RcAP2v* (i.e., two DREB1B, one AGL3, and one SPL14) (Supplementary Table S2). Additionally, we identified one miR172-binding site in the 10th exon of *RcAP2*, and one miR5021-binding site in the retained first intron of *RcAP2v* (**Figures 1D,E**). We designed the primers in the first intron-retaining region to detect the abundance of *RcAP2v*. However, the expression of *RcAP2v* was very low compared with *RcAP2* (**Figure 1F**). The sequences of miR172-binding site and miR5021-binding site were complementary to *RcAP2* sequences (**Figure 1G**). An analysis of the RcAP2-green fluorescent protein (GFP) fluorescence indicated that RcAP2 was localized to the nucleus of maize protoplasts (**Figure 1H**).

### Temporospatial Expression of *RcAP2* in *Rosa chinensis*

**FIGURE 2:**
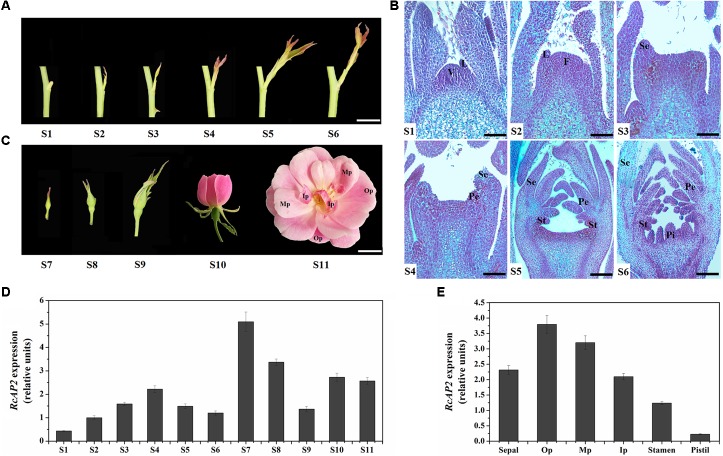
Spatiotemporal pattern of *RcAP2* expression in ‘Old Blush’ flowers. **(A)** Diagram of S1–S6 developmental stages of ‘Old Blush’ shoot. S1: stage 1, vegetation cone and leaf primordia formation; S2: stage 2, floral meristem formation; S3: stage 3, sepal primordia appear; S4: stage 4, petal primordia appear and sepal primordia continue to develop; S5: stage 5, stamen primordia appear and develop; S6: stage 6, pistil primordia appear and other types of primordia continue to develop. Bars = 10 mm. **(B)** Observation of S1–S6 paraffin sections. V, vegetation cone; L, leaf primordia; F, floral meristem; Se, sepal primordia; Pe, petal primordia; St, stamen primordia; Pi, pistil primordia. Bars = 80 μm (S1, S2), 120 μm (S3, S4), or 200 μm (S5, S6). **(C)** Phenotypes of S7–S11 developmental stages of ‘Old Blush’ flowers. S7: stage 7, small buds appear with diameter about 2 mm; S8: stage 8, small buds with diameter about 4 mm; S9: stage 9, bud differentiation is complete and sepals have not cracked; S10: stage 10, flower is partially open; S11: stage 11, flower is fully open, carpels and stamens appear. Op, outer whorl petals; Mp, middle whorl petals; Ip, inner whorl petals. Bars = 10 mm. **(D)** qPCR analysis of *RcAP2* transcript levels in 11 developmental stages of rose flower. S1–S11 corresponds to stages in **A–C**. **(E)** qPCR analysis of *RcAP2* transcript levels different floral organs and Op, Mp, and Ip as defined in **C**.

We divided the rose flower development process into 11 stages (S1–S11). Stages S1 to S6 comprise the main period of floral organ differentiation, during which elongating shoots begin to appear (**Figure 2A**). The initiation of floral organs and the formation of various primordia were observed at each stage using paraffin sections (**Figure 2B**). The primordia developed as follows. The vegetation cone and leaf primordia formed (S1). The vegetation cone was converted to the floral meristem (S2) and the outermost sepal primordia formed (S3). This was followed by the formation of the inner petal primordia (S4), stamen primordia (S5), and pistil primordia (S6). During S7 and S8, small buds became visible, while the internal floral organs continued to differentiate and develop. The flower buds were fully developed at S9, and started to bloom (**Figure 2C**).

*RcAP2* was expressed during all *R. chinensis* floral development stages. However, the *RcAP2* expression level was relatively low from S1 to S6 before rapidly increasing and peaking at S7. The *RcAP2* expression level gradually decreased as buds expanded. The opening of flowers was accompanied by an increase in *RcAP2* expression (**Figure 2D**). The distribution of *RcAP2* expression in different floral organs exhibited some regularity. We defined the first whorl with five petals as Op (i.e., outer whorl petals), the narrow petals closest to the stamens as Ip (i.e., inner whorl petals), and the remaining petals as Mp (i.e., middle whorl petals) (**Figure 2C**). *RcAP2* was most highly expressed in the Op. The *RcAP2* expression level gradually decreased from the Op to the stamen, with the lowest level detected in the pistil (**Figure 2E**).

### *RcAP2* Expression Was Associated With Changes in the Number of Rose Petals at Low and High Temperatures

**FIGURE 3:**
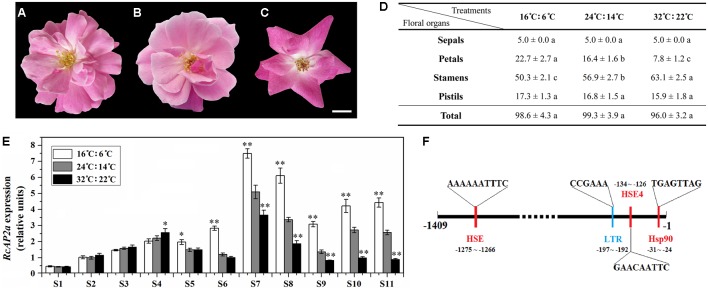
Effect of temperature on petal number of ‘Old Blush’ flowers. **(A–C)** Phenotypes of rose flowers at S11 under different temperatures. All buds were observed from beginning of S1. **(A)** 16°C: 6°C (light: dark). **(B)** 24°C: 14°C (light: dark). **(C)** 32°C: 22°C (light: dark). Bar = 1 cm. **(D)** Statistics of number of S11 floral organs under different temperatures. Values are means + SD of three biological replicates. Different lowercase letters indicate significant differences (Fisher’s LSD, *P* < 0.05). **(E)** *RcAP2* transcript levels in rose flowers in response to different temperature treatments. *RcAP2* transcript levels under 16°C: 6°C (light: dark) (white columns), under 24°C: 14°C (light: dark) (gray columns), and under 32°C: 22°C (light: dark) (black columns). Flowers from S1 to S11 were collected and analyzed. Values are means + SD of three biological replicates. ^∗^ and ^∗∗^ represent significant differences at *P* < 0.05 and *P* < 0.01, respectively (Student’s *t*-test). Flowers grown under 24°C: 14°C (light: dark) served as control. **(F)** Analysis of *cis*-acting elements in *RcAP2* promoter using PlantCARE (http://bioinformatics.psb.ugent.be/webtools/plantcare/html/) and SoftBerry (http://linux1.softberry.com/berry.phtml?topic=nsitep&group=programs&subgroup=promoter). Locations and sequences of four *cis*-acting elements that respond to temperature are shown: red, responsive to high temperature; blue, responsive to low temperature.

The temperatures ‘Old Blush’ plants are exposed to during cultivation influence the number of petals that are produced, with more petals at low temperatures. To characterize this phenomenon, we analyzed plants exposed to different temperatures. Similarly growing 2-year-old rose plants were incubated under one of the following conditions: 16°C (light): 6°C (dark); 24°C (light): 14°C (dark); or 32°C (light): 22°C (dark). The resulting phenotype and number of floral organs were analyzed (**Figures 3A–D**). There were no significant differences in the total number of floral organs among the three treatments. However, the flowers that underwent the low-temperature treatment (16°C: 6°C) produced more petals than the flowers exposed to normal temperatures (24°C: 14°C), while the flowers that underwent the high-temperature treatment (32°C: 22°C) produced fewer petals. The number of stamens exhibited the opposite pattern (**Figure 3D**). The excess petals were slender, and differed from the Op petals (**Figure 3A**). We also observed variability in the number of petals derived from stamens at different temperatures.

We analyzed the *RcAP2* expression level at all floral development stages under three temperature conditions (**Figure 3E**). The expression level under the normal temperature treatment (24°C: 14°C) was used as the control. The *RcAP2* mRNA level under the control conditions increased starting at S6, peaking at S7. Additionally, the low-temperature treatment was associated with the highest *RcAP2* expression levels from S5 onwards. The high-temperature treatment inhibited the increase in *RcAP2* expression from S7 until the flower blooming stage. We analyzed the *RcAP2* promoter sequence using the PlantCARE database and SoftBerry program. The promoter contained four *cis*-acting elements related to temperature responses, three heat shock elements, and one low-temperature response element (**Figure 3F** and Supplementary Table S3).

### Manipulation of *RcAP2* Ectopic Expression Modulated the Ratio of Petals to Stamens in *Arabidopsis thaliana*

**FIGURE 4:**
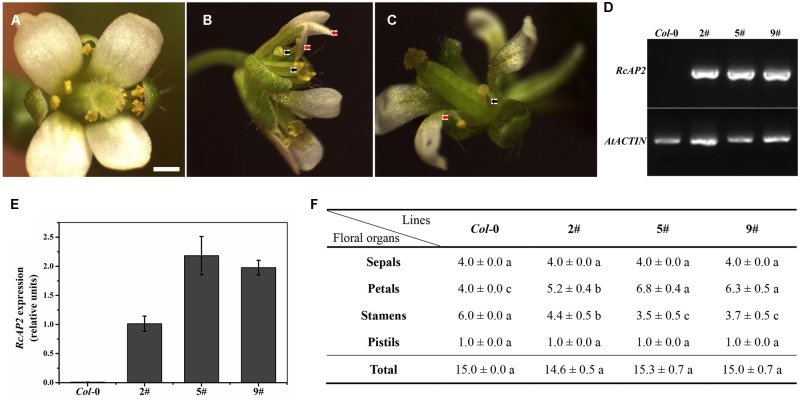
*RcAP2* ectopic expression in *Arabidopsis thaliana*. **(A)** Flower of wild-type *Arabidopsis thaliana* (Columbia). Bar = 0.5 mm. **(B)** Flower phenotype of 5# and 9# transgenic lines of *RcAP2*. **(C)** Flower phenotype of 2# transgenic line of *RcAP2*. **(B,C)** Red arrows indicate abnormal stamens resembling slender petals; black arrows indicate abnormal stamens with shortened filaments, flat anthers, and no pollen attached, similar to transitional-state stamens. **(D)** RT-PCR detection of *RcAP2* transcript levels in *Arabidopsis* transgenic lines. Wild-type *Col*-0 served as control. PCR-amplified *RcAP2* fragment was 418 bp (base pair) and *AtACTIN* fragment was 373 bp. The original gel image of **(D)** was provided in Supplementary Figure S2. **(E)** qPCR detection of *RcAP2* transcripts in *Arabidopsis* transgenic lines. Wild-type *Col*-0 served as control. **(F)** Statistics of floral organ numbers in wild-type and *Arabidopsis* transgenic lines. Values are means + SD of three biological replicates. Different lowercase letters indicate significant differences (Fisher’s LSD, *P* < 0.05).

We transformed *A. thaliana* (Columbia) plants with the *35S*::*RcAP2* plasmid to generate transgenic plants over expressing *RcAP2*. Transgenic lines in which *RcAP2* was highly expressed were identified by a reverse transcription polymerase chain reaction (RT-PCR) assay. We selected lines 2, 5, and 9 for subsequent analyses (**Figure 4**). The second whorl of line 5 flowers was obviously different from that of the wild-type flowers (**Figure 4B**). The number of petals increased from four (i.e., normal) to about seven, while the number of stamens decreased. Some stamens developed abnormally. Some formed as slender petals, while some formed as shortened filaments and anthers with no pollen attached, similar to transitional-state anthers. The slender petals were counted as petals. Abnormal stamens were also detected in line 2 (**Figure 4C**). There were almost no differences between the wild-type and transgenic plants regarding the total number of floral organs, including the number of sepals and pistils. The increase in the number of petals was offset by the decrease in the number of stamens (**Figure 4F**). The ectopic expression of *RcAP2* in *A. thaliana* may induce the conversion of stamens to petals, with no changes to the total number of floral organs.

### Silencing *RcAP2* by RNA Interference Decreased the Number of Petals in ‘Old Blush’ Flowers

**FIGURE 5:**
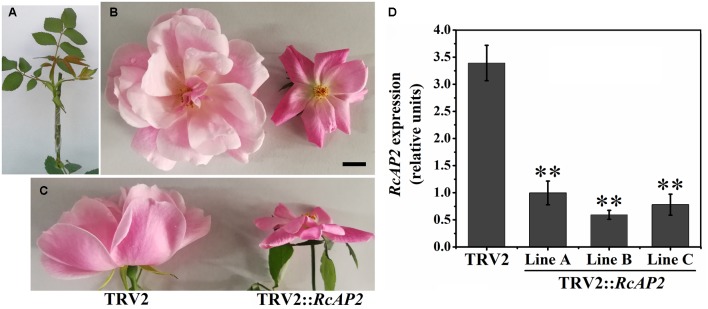
*RcAP2* gene silencing in ‘Old Blush.’ **(A)** Shoots surviving after infection with *A. tumefaciens* and grafting. **(B,C)** Flower phenotype of control (left) and *RcAP2*-silenced (right) flowers. **(C)** Side view of flowers. TRV2 represents *A. tumefaciens*-mediated insertion of pTRV1 + pTRV2 (control); TRV2::*RcAP2* represents pTRV1 + pTRV2::*RcAP2*. Bar = 1 cm. **(D)** qPCR detection of *RcAP2* transcript levels in VIGS-treated flowers. Gray bars represent *RcAP2* transcript levels relative to *RcActin* transcript levels. Lines A, B, and C represent three mixed samples, each consisting of four phenotypically obvious flowers. Values are means + SD of three technical replicates. ^∗∗^*P* < 0.01, (Student’s *t*-test).

The *R. chinensis RcAP2* gene was functionally characterized by virus-induced gene silencing (VIGS) using a graft-accelerated method (**Figure 5A**). The pTRV1 + pTRV2::*RcAP2* treatment resulted in an obvious decrease in the expression of *RcAP2*. Moreover, the number of petals decreased in the resulting flowers (**Figures 5B,C**). The outer whorl petals were preserved, the missing petals were petals from stamen origin. Compared with the effects of the pTRV1 + pTRV2 treatment control, the changes in *RcAP2* expression and the phenotype induced by the pTRV1 + pTRV2::*RcAP2* treatment confirmed that *RcAP2* had been partially silenced (**Figure 5D**). Additionally, the *RcAP2* transcript level was directly related to the number of petals in ‘Old Blush’ flowers.

## Discussion

To elucidate the functions of *R. chinensis AP2*, we analyzed its sequence in detail. We observed that *RcAP2* contains two AP2 domains, and exhibits characteristics typical of *AP2*. However, our phylogenetic analysis revealed the considerable genetic distance between the *AP2* of Rosaceae species and that of other plant species. Thus, *AP2* may exhibit different functions in different species. The subcellular localization of RcAP2 indicated this protein is present in the nucleus, which is consistent with the localization of transcription factors. In addition to the 3′ region, there are many miRNA-binding sites located in the 5′ and CDS regions for inhibiting translation (Hausser et al., 2013). We predicted the presence of a miR172-binding site in the CDS region of *RcAP2*. Recent studies concluded that miRNAs are important for the development of plant floral organs. The binding of miRNAs to specific sites in the target mRNA sequences affect floral development and regulate floral organ traits (Jones-Rhoades et al., 2006). Additionally, miR172 not only controls *AG* expression, it also restricts *AP2* expression in the third and fourth whorls of floral organs during the early flowering stages, degrades *AP2* mRNA, and inhibits *AP2* translation. A previous study revealed that miR172 and its *AP2* target are highly conserved components related to the regulation of plant development (Zhu and Helliwell, 2010). However, another study concluded that the regulatory activities of miR172 are insufficient for explaining the contradictory relationship between *AP2* expression and its genetic activity (Wollmann et al., 2010). We also detected a miR5021-binding site in the retained intron of *RcAP2v*, but the function of miR5021 in plants is unknown. The translation of *RcAP2v* ends in the first intron, and the generated protein does not contain two AP2 domains. The variable splicing of different exons sometimes results in the early termination of a protein, and may function as a molecular switch (Kelemen et al., 2013). Thus, *RcAP2v* may contribute to the regulation of *RcAP2* activity, although, it has a very low expression at the normal temperature. Temperature-dependent alternative splicing has been first described for the MADS-box gene *FLM* involved in the control of flowering time (Posé et al., 2013), and further identified for a large number of genes (Verhage et al., 2017). *RcAP2* appears as a likely candidate to have an alternative splice variant whose levels depend on temperature which needs further study. Our sequence analyses sugges*t RcAP2* may be functionally complex.

In *A. thaliana*, *AP2* mRNA is distributed throughout various types of floral primordia and vegetative tissues (e.g., stems and leaves). Additionally, the *AP2* promoter is active in all floral whorls (Jofuku et al., 1994; Würschum et al., 2006). The *AP2* orthologs in petunia, apple, and strawberry are also ubiquitously expressed (Maes et al., 2001; Su et al., 2004; Shengmei et al., 2006). Although *AP2* is widely expressed, there are obvious differences in mRNA accumulation among floral parts. The *AP2* orthologs exhibit very specific expression patterns in the floral primordia and inflorescences of maize and snapdragon (Keck et al., 2003; Chuck et al., 2008). In *A. thaliana*, *AP2* mRNA accumulates predominantly in the outer floral whorls (Wollmann et al., 2010). We observed that *RcAP2* expression in each floral organ whorl underwent changes as flowers developed. It is noteworthy that *RcAP2* expression increased significantly from S6 to S7. This is the period in which the primordia of all floral organs are formed, but they have not developed into their corresponding floral organs. We speculate that floral organ development is affected by many factors, including intrinsic gene regulatory activities as well as environmental stimuli. Petals from stamen origin are the main source of rose petals. An increase in the petaloid nature of stamens was associated with a gradual transition of the stamens to petals, along with upregulated *RcAP2* expression. These results imply that *RcAP2* may be involved in the development of petals from stamen origin in *R. chinensis*.

Previous studies uncovered the changes in the number of rose petals induced by low temperatures (Zieslin, 1968, 1992). Additionally, low temperatures also modify the methylation status of a specific region in the *RhAG* promoter (Ma et al., 2015). In our study, the number of petals from stamen origin increased and decreased in response to low- and high-temperature treatments, respectively. The *RcAP2* expression level was also affected by temperature changes. In *A. thaliana*, one model for the antagonistic relationship between class A and C genes involves *AP2* and *AG* during the regulation of perianth formation. Additionally, *ap2* mutants reportedly exhibit enhanced *AG* activity, and the outer floral organs develop into reproductive organs (Bowman et al., 1991). However, in stamen primordia, *AP2* and *AG* expression overlap. Additionally, *AP2* mRNA can be detected in all four whorls of the *ag* mutant, implying *AG* does not regulate *AP2* at the transcript level (Bomblies et al., 1999; Wollmann et al., 2010). Some *35S::AP2 Arabidopsis* transgenic lines with more stamens than the wild type, and in *35S::AP2m1* lines, a mutant with six mismatches to miRNA172, presented the transformation of stamens to petals, which was consistent with the ability of *AP2* to negatively regulate *AG* (Chen, 2004). In *A. thaliana*, *AP2* can bind the non-canonical AT-rich target sequence, TTTGTT, in the second intron of *AG* to directly regulate *AG* expression (Dinh et al., 2012). We analyzed the full-length *RcAG* sequence and detected three TTTGTT sequences in the first intron (Supplementary Figure S1). These conserved target sequences may represent a link between *RcAP2* and *RcAG*.

In *A. thaliana*, the *ap2* mutants exhibit structural deformities in the four whorls of floral organs. Specifically, whorl 1 is transformed into pistils, there is a decrease in the number of whorls 2 and 3, and the carpels do not fuse (Jofuku et al., 1994). Furthermore, transgenic *Nicotiana benthamiana* plants expressing the *A. thaliana* miR172-resistant *AP2* produce flowers with an abnormal number of petals and stamens (Mlotshwa et al., 2006). Meanwhile, the expression of the water lily *AP2* homolog in *A. thaliana* plants leads to a significant increase in the number of petals (Luo et al., 2012). A previous study confirmed that *AP2* can promote ectopic floral organ formation in a process that may depend at least partially on a stem cell transcription factor, WUS (Zhao et al., 2007). In the current study, we observed a decrease in the number of stamens as well as changes in stamen morphology in *RcAP2*-expressing transgenic *A. thaliana* plants. This suggests the *R. chinensis AP2* homolog inhibits the expression of class C genes and limits the tissues in which these genes are expressed, ultimately disrupting the third whorl of floral organs. The ectopic expression of *RcAP2* is accompanied by the production of abnormal stamens, leading to an increase in the number of petals, which is similar to the effect of low temperatures on *R. chinensis* flowers. Silencing *RcAP2* in ‘Old Blush’ plants via VIGS decreased the number of petals derived from stamens, but did not affect the five petals of the outermost whorl. Therefore, the *RcAP2* transcript level is directly related to the number of petals (i.e., the number of petals derived from stamens). Temperature-induced changes to *RcAP2* expression and the ectopic expression of *RcAP2* in *A. thaliana* or *RcAP2* silencing in rose (i.e., human manipulation) alter the number of petals in flowers. The presence of temperature-related *cis*-acting elements in the *RcAP2* promoter also serves as evidence that *RcAP2* expression is responsive to temperature changes. The temperature-induced phenotypic changes to rose flowers are very important for the ornamental flower industry, and are associated with the differentiation and development of rose floral organs. Our results suggest that *RcAP2* may be an important regulator of the number of petals, and may also mediate the temperature-related formation of petals derived from stamens.

## Author Contributions

QZ and YH conceived and designed the study. YH and AT performed most of the experiments. YH analyzed the data and wrote the manuscript. HW, TZ, TC, JW, WY, and HP provided help with the experiments and data analysis.

## Conflict of Interest Statement

The authors declare that the research was conducted in the absence of any commercial or financial relationships that could be construed as a potential conflict of interest.

## Footnotes

1 http://www.ncbi.nlm.nih.gov/genbank/

2 http://www.ncbi.nlm.nih.gov/projects/gorf/

3 http://planttfdb.cbi.pku.edu.cn/prediction.php

4 http://regrna2.mbc.nctu.edu.tw/detection.html

5 http://bioinformatics.psb.ugent.be/webtools/plantcare/html/

6 http://linux1.softberry.com/berry.phtml?topic=nsitep&group=programs&subgroup=promoter
