# Supplementary material for: An APETALA2 Homolog, RcAP2, Regulates the Number of Rose Petals Derived From Stamens and Response to Temperature Fluctuations
Source: Front Plant Sci. 2018 Apr 12;9:481. doi: 10.3389/fpls.2018.00481 (PMC5906699; doi:10.3389/fpls.2018.00481)
Supplement: Supplementary file 4 [file Table_4.DOCX]

Supplementary Material

**An *APETALA2* homolog, *RcAP2*, regulates the number of rose petals derived from stamens and response to temperature fluctuations**

Yu Han^1^, Aoying Tang^1^, Huihua Wan^1^, Tengxun Zhang^1^, Tangren Cheng^1^, Jia Wang^1^, Weiru Yang^1^, Huitang Pan^1^ & Qixiang Zhang^1, 2*^

*** Correspondence:** Qixiang Zhang, email: zqxbjfu@126.com

1 Beijing Key Laboratory of Ornamental Plants Germplasm Innovation & Molecular Breeding, National Engineering Research Center for Floriculture, Beijing Laboratory of Urban and Rural Ecological Environment, Key Laboratory of Genetics and Breeding in Forest Trees and Ornamental Plants of Ministry of Education, School of Landscape Architecture, Beijing Forestry University, Beijing, 100083, China

2 Beijing Advanced Innovation Center for Tree Breeding by Molecular Design, Beijing Forestry University

**Supplemental information:**

Supplemental Table S1: The exons information of *RcAP2* DNA sequence.

Supplemental Table S2: The transcriptional regulatory motifs information of *RcAP2* cDNA sequence.

Supplemental Table S3: The cis-acting elements information of *proRcAP2*.

Supplemental Table S4: The primer sequences involved in this article.

Supplemental Figure S1: The AT-rich sequence element analysis of *RcAG* DNA sequence.

Supplemental Figure S2: The original image from Figure 4D.

Supplemental Table S4: The primer sequences involved in this article.

| **Primer name** | **Primer sequence** |
| --- | --- |
| *RcAP2*-F1 | ACAACAGCCGATGAAGAAG |
| *RcAP2*-R1 | AATTGCATAGAGGCAGACG |
| 5' GSP | TGCAGTCCCAAAAGCCGGGTTATTCTT |
| 3' GSP | GAGTGAGCTGAACTCAACTGAGCCTTCT |
| UPM | CTAATACGACTCACTATAGGGCAAGCAGTGGTATCAACGCAGAGT |
| *RcAP2*-F2 | ATGTGGGATCTCAATGACTCT |
| *RcAP2*-R2 | CTAGCGAGAGTGGAACCCAT |
| *RcAG*-F | ATGGCCTATGAAAACAAACCC |
| *RcAG*-R | TCAAACTAATTGAAGGGAAATCTGG |
| *proRcAP2*-SP1 | CCTCACTGGTCTCTCTAACTCAACAC |
| *proRcAP2*-SP2 | CAACCCCAGAATTGTTCCTCGCAAC |
| *proRcAP2*-SP3 | GCTCGAAGATCTATTGATGTATGGG |
| qPCR-*RcActin*-F | GGGTTTGACACATCTTGCC |
| qPCR-*RcActin*-R | TTCGGATTCATCGTCCAC |
| qPCR-*AtActin*-F | GGTATGGGTCAGAAAGATGCT |
| qPCR-*AtActin*-R | CGTTGTAGAAAGTGTGATGCC |
| qPCR- *RcAP2*-F | CCCAAGCCTAGAGAGGATAATG |
| qPCR- *RcAP2*-R | GGGATGGAGATTGGAGGTTT |
| qPCR- *RcAP2v*-F | TGATTCAGGGATTGCGG |
| qPCR- *RcAP2v*-R | CTGTTTCAAGTCTTCTTCATAGTCG |
| *RcAP2*-F3 | GGGGCCCGGGGTCGACATGTGGGATCTCAATGACTCTCCT |
| *RcAP2*-R3 | TACCGGATCCACTAGTGCGAGAGTGGAACCCATTA |
| RT-*RcAP2*-F | GCTTCGCCTTCTTCTTCTTT |
| RT-*RcAP2*-R | CCTCCCATCTTCCACACTT |
| RT-*AtActin*-F | ATGGTTGGTATGGGTCAG |
| RT-*AtActin*-R | CCTCGTAGATTGGCACAGT |
| *RcAP2*-F4 | ATGTGGGATCTCAATGACTCTCCT |
| *RcAP2*-R4 | GCGAGAGTGGAACCCATTA |
| pTRV2-*RcAP2*-F | TAAGGTTACCGAATTCGCGATCAAGTTCAGGGGAGTA |
| pTRV2-*RcAP2*-R | GCTCGGTACCGGATCCTCCCAAACTCAAATCCAGATTG |
